# A Pilot Goal-Oriented Episodic Future Thinking Weight Loss Intervention for Low-Income Overweight or Obese Young Mothers

**DOI:** 10.3390/nu15133023

**Published:** 2023-07-03

**Authors:** Mei-Wei Chang, Alai Tan, Duane T. Wegener, Rebecca E. Lee

**Affiliations:** 1 College of Nursing, The Ohio State University, 1585 Neil Avenue, Columbus, OH 43210, USA; tan.739@osu.edu; 2 Department of Psychology, The Ohio State University, 1835 Neil Avenue, Columbus, OH 43210, USA; wegener.1@osu.edu; 3 Center for Health Promotion and Disease Prevention, Edson College of Nursing and Health Innovation, Arizona State University, 550 N. 3rd St., Phoenix, AZ 85004, USA; releephd@yahoo.com

**Keywords:** autonomous motivation, self-efficacy, stress, emotion control, low-income women, obesity, weight loss

## Abstract

Background. Episodic future thinking (EFT) has shown efficacy in laboratory settings. We conducted a pilot goal-oriented EFT (GoEFT) intervention in a real-world setting to help low-income overweight or obese mothers lose weight. This paper presents intervention acceptability and efficacy. Methods. The study used a single-group, before–after design. During the 3-week intervention, participants (N = 15) completed weekly web-based lessons and online health coaching sessions to manage stress and emotion, eat healthier, and be more physically active. Participants completed online surveys at baseline and immediately after the intervention. They also completed an interview to evaluate intervention acceptability. We applied paired t-tests to evaluate efficacy and used content analysis to discover interview themes. Results. Participants consistently identified the intervention as acceptable, noting the usefulness of pre-written goals, GoEFT strategies, and goal progress evaluations. The intervention effectively promoted weight loss (*d* = −0.69), fruit and vegetable intake (*d* = 0.45–0.49), and emotion control (*d* = 0.71). It also reduced fat (*d* = −0.51) and added sugar intake (*d* = −0.48) and alleviated stress (*d* = −0.52). Moreover, the intervention increased autonomous motivation (*d* = 0.75–0.88) and self-efficacy (*d* = 0.46–0.61). Conclusion. The GoEFT intervention was acceptable to participants, showing strong preliminary efficacy.

## 1. Introduction

In the U.S., overweight and obesity disproportionately affect low-income women of child-bearing age [1], a significant public health problem for many reasons. Approximately 40–50% of obese women gain ≥2 body mass index (BMI) units between pregnancies [2]. When overweight or obese women become pregnant again, they are at high risk for adverse maternal birth outcomes (e.g., gestational diabetes, large baby at birth) partly due to excessive gestational weight gain [3,4] and pre-pregnancy overweight or obesity [5,6,7]. Compared with normal-weight women, overweight or obese women are at increased risk (≥3.2-fold) of major weight retention (>20 lbs) at 1 year postpartum [2,8], leading to lifelong obesity and its associated chronic conditions.

Healthy lifestyle behaviors promote weight loss, effectively reducing the risk of obesity-related chronic conditions, for example, type 2 diabetes and cardiovascular disease [9,10]. However, weight loss challenges individuals. To lose weight, individuals have to be able to plan and engage in successful weight loss strategies (for example, goal setting, self-monitoring), which require a higher order of cognition or executive function [11]. Executive function includes aspects such as working memory, inhibitory control, cognitive flexibility, planning, reasoning, and problem solving. Such abilities enable individuals to set and keep a goal in mind, apply learned strategies to accomplish the goal, problem solve, think creatively, stay focused, know what to inhibit or resist (temptation), and view situations from different perspectives [12]. Executive function is associated with motivation (autonomous motivation, reflecting personal values and interest, and self-efficacy) [13] and affect (stress and emotion) [12,14,15,16], all of which are associated with adherence to healthy lifestyle behaviors and weight loss [17,18,19,20,21]. Yet, no prior lifestyle behavioral weight loss intervention studies have integrated executive function, motivation (autonomous motivation and self-efficacy), and affect (stress and emotion) to promote weight loss.

A promising approach to integrate the above-mentioned concepts is through goal-oriented episodic future thinking (GoEFT) [22,23,24,25,26], vividly imagining oneself in the future to pre-experience upcoming goal-related activities (picturing) [27,28]. Episodic future thinking improves executive function, motivation, and affect [29,30,31,32,33,34,35,36,37], but tests have been limited to laboratory settings [22,24,25] with the exception of a recent field intervention study applying GoEFT to promote weight management in overweight or obese pregnant women [38].

We conducted a pilot GoEFT intervention to promote weight loss in low-income overweight or obese mothers with young children. The pilot intervention applied concepts of executive function, autonomous motivation, and self-efficacy to promote stress and emotion management, healthy eating, and physical activity. The objective of the study was to evaluate the feasibility of intervention implementation by assessing intervention acceptability to the participants. This paper presents the intervention acceptability and efficacy of the GoEFT intervention.

## 2. Materials and Methods

### 2.1. Setting and Participants

This study used a single-group, before–after design. We recruited participants through Facebook, flyers posted in local community settings, MyChart (a secure website that gives individuals access to their health information), and Research Match (a national network that identifies potential participants for available studies). Participants were recruited and enrolled between July 2021 and March 2022. Women who were interested in the study emailed the study office and were screened over the phone. To be qualified to participate, women had to have been currently enrolled in government assistance programs (for example, the Special Supplemental Nutrition Program for Women, Infants, and Children, Supplemental Nutrition Assistance Program, Medicaid), between 6 weeks and 5 years postpartum, 18–45 years old, and fluent in speaking, reading, and writing English. Also, they had to own a smart phone with unlimited text messages and internet access, commit to a 3-week intervention study, and have a BMI (calculated using self-reported height and weight) between 25.0 and 44.9 kg/m^2^. Women were excluded if they were pregnant, planned to become pregnant during the trial, had diagnoses of type 1 or 2 diabetes, untreated thyroid disease, major psychiatric disorder (e.g., schizophrenia, bipolar), a history of bulimia or anorexia, drug or alcohol abuse or dependence within the last 6 months, had taken appetite suppressant or antipsychotic medications known to affect body weight or were currently participating in a weight control or drug study, were currently participating or planning to participate in a commercial weight loss program, or had undertaken previous weight loss surgery or had contraindications to physical activity.

Eligible women received the informed consent document through email to review prior to attending the scheduled Zoom 1 meeting (information session) where women provided electronic consent via REDCap. After providing consent, women completed the baseline survey via REDCap and were invited to attend a Zoom 2 meeting to be enrolled in the study. Survey data were collected at 2 time points: baseline and immediately after the 3-week intervention. The study procedure was approved by the Ohio State University Institution Review Board (IRB # 2020B0322).

### 2.2. Intervention: Web-Based Intervention and Online Health Coaching

The 3-week intervention was delivered via a weekly web-based intervention through Qualtrics and individual health coaching. The web intervention with written text only was developed by working closely with 5 low-income overweight or obese mothers with young children who were peer advisors for our previous intervention study [39]. Our prior study aimed at helping overweight or obese mothers with young children prevent further weight gain through stress management, healthy eating, and physical activity [39]. Although the intervention had no overall effect on body weight [39], intervention participants reported eating less high-fat food [40], being more physically active [41], and feeling less stress and depressive symptoms [42] than the comparison group. Briefly, we applied strengths and addressed lessons learned from our previous intervention study [39]. Examples of strengths included providing easy and practical strategies that participants could easily apply to daily life to manage stress and emotion, eat healthier, and be more physically active, addressing the common daily challenges, and reminding participants about receipt of benefits. Examples of lessons learned included the need to guide participants to make plans to accomplish their goals and the need to provide pre-written goals because participants had challenges in generating goals [39]. The 3-week intervention topics included stress and emotion management, healthy eating, and physical activity (one topic per week). Each topic included 6–12 pre-written goals (total of 32 pre-written goals) with associated specific and realistic plans for accomplishing the goals. These goals were created to address common daily stressors (for example, laundry, dishes, taking care of young children), causes of negative emotion (for example, self-blame), and desire to be a good role model, eat healthier, and be more physically active with young children. Example goals were doing laundry, drying clothes, and putting them away within a day, reducing junk food intake, and being physically active with young children. Accompanying each goal, we provided examples of the study concept application. To boost autonomous motivation, participants identified a personal value followed by assessing the congruency between the chosen value and current behaviors. To increase self-efficacy, they identified their past successful strategies (e.g., positive self-talk) for accomplishing the goal. To increase executive function, participants identified immediate and long-term pros and cons for making or not making positive changes. They were also asked to keep the goal and personal value in mind and apply newly learned information to overcome barriers to implement plans for accomplishing the goal.

Prior to enrollment, we carefully piloted the intervention with several peers who were not involved in the intervention development or enrolled in the study. These peers provided feedback to help us finalize the intervention. The web intervention had 2 parts (total of 30 min/week): Parts I (25 min/week) and II (5 min/week). Each week, we sent 2 reminders with the intervention link (days 1 (Part I) and 5 (Part II), via text and email) to remind participants to complete the web intervention. After using their unique username and password to log in, participants used dropdown menus to select options (type in available) except for the section asking them to make specific plans for accomplishing their chosen goals (described below). Day 1: Web Part I. First, participants identified a personal value, ways to increase confidence, short- and long-term benefits for making positive changes, and potential barriers to implementing plans and associated strategies to overcome the chosen barriers to implementing plans for accomplishing goals. Next, they pictured the selected options. After that, they selected 1 pre-written goal to meet their needs for the week. To help participants generate plans, we provided a web link with example plans associated with the chosen goal. Then, participants recorded (typed in) WHAT the goal-fulfilling activity was, WHY it would serve personal values and goals, WHERE, WHEN, and with WHOM the activity would take place, and HOW it could be accomplished. Finally, participants pictured their plans for accomplishing goals. Participants were asked to picture their value, goal, pros and cons for making or not making positive changes, and plans for accomplishing the goal daily. Daily picturing is an effective strategy to increase motivation to implement plans for accomplishing chosen goals [43]. Days 2–5: Online Health Coaching. The online health coaching sessions were scheduled at a convenient time for participants and were recorded with the participants’ permission. During each session, the health coach reviewed the chosen activities (in Web Part I) and evaluated the participant’s plan for accomplishing the goal. The health coach provided example plans (from the list already included in Part 1) as needed if participants did not have specific and realistic plans for accomplishing their goals. During each session, participants engaged in picturing a personal value, the week’s goal, pros and cons for making or not making changes, and plans for accomplishing the chosen goal. The health coach ended the session by reminding participants to continue picturing at least 2 times per day. Days 6–7. Web Part II. Participants logged in to the intervention website to evaluate goal progress followed by receiving a tailored message (an affirmation statement). Next, participants used dropdown menus to choose which benefits they received for making positive changes.

### 2.3. Measures

Acceptability evaluation. The trained interviewers used semi-structured interview questions (Table 1) to conduct an individual interview with each participant (~30 min) through Zoom to evaluate the acceptability of the intervention (web intervention and online health coaching). Of the 12 participants who completed the study, 11 were interviewed. The interviews were recorded with participants’ permission. All interviews were transcribed verbatim; transcriptions were verified for accuracy.

Body weight and BMI. Participants self-reported body weight in pounds. The self-reported height and weight were used to compute BMI (kg/m^2^).

Percent energy from fat. To measure percent energy from fat, we used the National Cancer Institute (NCI) percent energy from fat survey (17 items) [44]. Participants rated the frequency of eating or drinking each specific food item using an 8-point scale: from “never” to “2 or more times per day”. We followed the procedure and algorithm provided by the NCI to calculate percent energy from fat [45]. Higher scores indicated higher percent energy from fat.

Added sugar intake. To measure added sugar intake, we used the NCI 5-factor screener to measure added sugar intake (4 items). Participants rated the frequency of drinking/eating a specific food item using a 10-point scale: from “never” to “5 or more times a week”. We followed the procedures provided by the NCI to estimate added sugar intake in teaspoons [46]. Higher scores meant higher added sugar intake.

Fruit and vegetable intake. To measure fruit and vegetable intake, we used the NCI 5-factor screener to measure fruit and vegetable intake (9 items). Participants rated the frequency of eating or drinking specific foods during the past month using a 10-point scale: from “never” to “5 or more times per day”. We followed the procedures and algorithm provided by the NCI to estimate individuals’ fruit and vegetable intake in cups [47]. Higher scores indicated more fruit and vegetable intake.

Physical activity. To measure physical activity, we used the short-form International Physical Activity Questionnaire (8 items) [48]. Participants were asked about 3 specific types of activity such as walking, moderate-intensity activity, and vigorous-intensity activity. Participants also reported frequency (days per week) and duration (time per day) for each specific type of activity. To create metabolic equivalent of task (MET, energy expenditure) measures, we multiplied frequency by duration (in hours). We then used the summation times 3.3 MET (walking), 4.0 (moderate physical activity), and 8.0 (vigorous physical activity) to generate physical activity scores in MET units [48]. Higher scores indicated more energy expenditure.

Stress. We used the Perceived Stress Scale (10 items) to measure stress. Participants rated the frequency of stressful life situations in the past month using a 4-point scale: 1 (never) to 4 (often) [49]. We summed responses to the 10 items to create a score for stress. Higher scores meant perceived higher levels of stress.

Emotional control. To measure emotional control, we used the Emotion Regulation Questionnaire to assess the emotional regulatory process using reappraisal (6 items) and suppression (4 items) [50]. Participants rated the degree of agreement using a 7-point scale: 1 (strongly disagree) to 7 (strongly agree). The suppression items were reverse coded prior to score computation. We summed responses to the 10 items to create a score for emotional control. Higher scores meant better emotional control.

Autonomous motivation. To measure autonomous motivation, we used the Treatment Self-Regulation Questionnaire [51] (18 items: 6 items for healthy eating, 6 items for physical activity, and 6 items for stress management). Participants rated how true each statement was for them related to healthier eating, physical activity, and stress management using a 7-point scale: 1 (not at all true) to 7 (very true). We averaged responses to the 6 items to create a score for healthy eating. The same approach was applied to create a score for physical activity and a score for stress management. Higher scores indicated higher autonomous motivation.

Self-efficacy. We used surveys to measure self-efficacy for healthy eating (8 items) [52] and physical activity (6 items) [53]. Participants rated levels of confidence using a 4-point scale: 1 (not at all confident) to 4 (very confident). We summed responses to the 8 items to create a score for self-efficacy for healthy eating. The same approach was applied to create a score for self-efficacy for physical activity. Higher scores meant higher self-efficacy. We also used the general self-efficacy scale (10 items) to measure self-efficacy. Participants rated the truthfulness of each statement using a 4-point scale: 1 (not at all true) to 4 (exactly true) [54]. We summed responses to the 10 items to create a score for general self-efficacy. Higher scores meant higher self-efficacy.

### 2.4. Data Analysis

Qualitative analysis. A codebook was created by reading the first 5 transcriptions line by line. We used a deductive process to identify the common themes [55]. Coding was compared between 2 research assistants, and discrepancies were discussed with the first author until reaching consensus.

Statistical analysis. Descriptive statistical analysis was applied to examine demographics. We used paired *t*-tests to evaluate the intervention effect by comparing the changes in each study variable from the baseline to immediately after the 3-week intervention. Cohen’s D (*d*) was used to report effect size. SAS 9.4 was used to perform all statistical analyses.

## 3. Results

**Figure 1:**
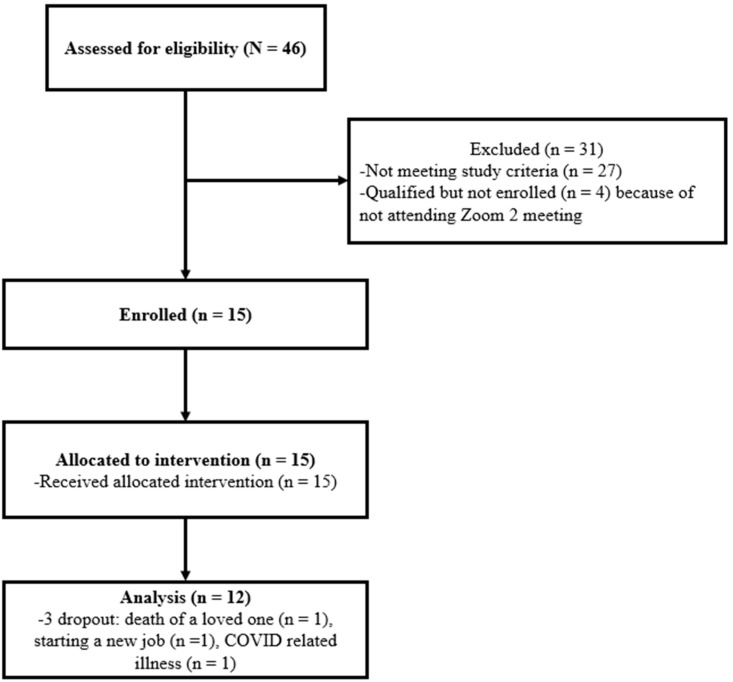
Recruitment flow chart.

Figure 1 presents a recruitment flow chart. The main reasons for not enrolling were BMI being outside the range of 25.0–44.9 kg/m^2^ and/or not being enrolled in any government assistance programs. Of the 15 women who enrolled, 7 were non-Hispanic Black, 5 were non-Hispanic White, 1 was non-Hispanic mixed race, and 2 were Hispanic. The average age of the women was 32.9 years (SD = 6.9). Eight women were single (never married), five were currently married, and two were divorced. All women had high school/GED or above education: one high school/GED, five some college or technical school but no degree, one associate’s degree, four bachelor’s degrees, and four master’s degrees. About half (8 out of 15) of the women were employed, with four being full-time employed, three being part-time employed, and one being self-employed.

### 3.1. Intervention Adherence

Two participants (2/15, 13.4%) dropped out immediately after completing the week 1 web part I intervention because of the death of a family member and starting a new job, respectively. An additional woman dropped out after completing the week 2 intervention due to COVID-19-related illness. These led to an 80% (12/15) retention rate at the end of the study (Figure 1). Of the 12 women who completed the study, everyone (100%) completed the three weekly web intervention activities (both parts I and II) and joined the three online health coaching sessions (100%).

### 3.2. Acceptability of Intervention

Table 2 presents common themes with quotes.

Regarding the appropriate intervention length, the need to extend the intervention period, and helpful reminders to engage in the web intervention, participants said that the length of web parts I and II was appropriate. Although some thought the length of each health coaching session was appropriate, others suggested breaking it down to twice a week to more easily fit into busy mothers’ schedules. Participants consistently requested extending the intervention period (>3 weeks). They also requested receipt of a summary for each of the web parts I and II. They said the reminders with the intervention link (through email and text) reminded them to engage in online intervention activities.

Regarding the usefulness of pre-written goals, participants unequivocally reported the usefulness of the pre-written goals because they believed it would be difficult for them to generate the goals on their own. They also said that the pre-written goals helped them stay focused, prioritize plans, think outside the box, and feel accountable for accomplishing their goals.

Regarding the usefulness of the 5Ws (WHAT, WHY, WHERE, WHEN, WHO) and HOW to make plans for accomplishing the chosen goal, participants consistently reported that the application of the 5Ws and HOW was useful to help them think through plans for accomplishing their goal for the week.

Regarding the helpfulness of example plans, participants valued that the health coach provided specific and realistic plans that worked for their peers to accomplish their goals. They noted that they found these plans to be easy, useful, and practical to implement in their daily lives. Participants also appreciated that the health coach shared common barriers to participants’ peers implementing plans. Consequently, they no longer felt alone or isolated because they realized that their situations were not unique but were common among their peers.

Regarding the benefits for engaging in goal progress evaluation, all participants consistently reported the usefulness of the weekly goal progress evaluation. They said this activity helped boost confidence, motivated them to make continued positive changes, and helped them to be accountable for accomplishing goals.

Regarding increasing motivation to implement plans through GoEFT, women unequivocally said they enjoyed picturing their weekly goals several times a day because it was quick and easy and highly motivated them to make and implement plans for accomplishing their goals.

Regarding positive intervention impact on children, confidence, and mental health, participants consistently reported that the intervention helped them think outside the box, view situations from different perspectives, stay focused, and prioritize plans. They also said that the study helped them realize the importance of taking care of themselves instead of always focusing on others. Moreover, they noticed an improvement in their relationships with children and felt much happier.

### 3.3. Intervention Effects

Table 3 presents pre- and post-intervention effects. Results showed that the intervention decreased mean body weight (*d* = −0.69), BMI (*d* = −0.77), percent energy from fat (*d* = −0.51), and added sugar (*d* = −0.048) and increased fruit and vegetable intake (*d* = 0.45–0.49). However, the intervention had a minimal effect on the promotion of physical activity (*d* = 0.13). The intervention relieved stress (*d* = −0.52) and increased emotional control (*d* = 0.71). Finally, the intervention increased autonomous motivation (*d* = 0.75 for healthy eating, *d* = 0.78 for physical activity, *d* = 0.88 for stress management) and self-efficacy (*d* = 0.61 for healthy eating, *d* = 0.46 for physical activity, *d* = 0.58 for general self-efficacy).

## 4. Discussion

This pilot study was conducted during the COVID-19 pandemic, and our intervention was not originally designed to take into consideration that unprecedented event. Despite those challenges, we successfully pilot tested the intervention. Our participants consistently evaluated the intervention favorably. Also, our intervention helped participants lose weight, eat more healthfully (less fat and added sugar and more fruits and vegetables), increase autonomous motivation and self-efficacy, alleviate stress, and better control emotions.

In line with the qualitative data related to our prior intervention with pregnant women [38], the current participants unequivocally said that the GoEFT (picturing) intervention was easy and quick to apply in daily life. Also, GoEFT helped them keep a personal value and goal in mind, thus increasing motivation to generate and implement plans for accomplishing their goals. Moreover, participants reported positive intervention impacts on their lives, for example, improving relationships with children.

A previous study showed that participants had challenges in goal setting [39]. Thus, we created pre-written goals, which were well received by low-income mothers with young children. This finding was consistent with our prior pilot intervention study of pregnant women [38]. Future studies might consider creating pre-written goals that better address the needs of the target audience. We observed (through listening to health coaching recordings) that low-income mothers had challenges in making specific and realistic plans for accomplishing their goals even though we provided a link with written example plans. It is interesting to observe that our participants reacted differently to the same or similar example plans presented in writing or by the health coach. None of the participants reported using the written example plans to make plans for accomplishing the pre-written goals. However, they unequivocally agreed that the example plans provided by the health coach were helpful. The coach consistently added a statement, such as “other mothers with similar life situations, found xyz helpful”. The above-mentioned observations occurred in our pilot pregnant women intervention study. Thus, future interventions might benefit from emphasizing the similarity of participants’ situations with those of peers who had successfully overcome those obstacles.

A prior 4-week pilot study using episodic future thinking to promote weight loss in parents of school-aged children showed that intervention participants lost 1 BMI (~7 lbs), a significantly greater amount of weight loss than in the comparison group (0.2 BMI, ~1.4 lbs) [26]. We applied GoEFT to promote weight loss among low-income overweight or obese mothers with young children. Our results revealed that participants lost 2.92 lbs over the 3-week intervention [22,56]. Results of prior work [26] and our own studies suggest that episodic future thinking can be a potentially promising approach for facilitating weight loss. Yet, intervention studies with a longer intervention period and longer-term follow-up are needed to validate these study findings.

We recently completed a separate pilot intervention study applying GoEFT to help overweight or obese pregnant women (N = 70) prevent excessive gestational weight gain in a real-world setting. Participants (N = 70) were randomized to a 20-week intervention or usual care group [38]. Results of dietary fat and added sugar intake between our prior pregnant women and current low-income women intervention studies were inconsistent. Though our pilot pregnant women intervention increased dietary fat and added sugar intake (at 35–37 weeks gestation, 4 weeks after the 20-week intervention) [57], the current intervention with low-income women who were not pregnant decreased fat and added sugar intake. Other than that, the results of both studies consistently revealed that the GoEFT intervention positively influenced fruit and vegetable intake, physical activity, stress, emotional control, autonomous motivation, and self-efficacy [57]. We are unable to compare the results of executive function because the current study did not measure it due to resource constraints.

There are study limitations. The one-group before–after study design hindered our ability to attribute the improvements to the intervention per se rather than the passage of time or any monitoring of health-related behaviors over the same time period. The absence of a non-interventional control group is a limitation of this work, and the next step in this line of work is to employ an experimental design to avoid this source of bias in the future. Yet, this pilot study was primarily designed to assess intervention acceptability by the study participants. Body weight data were collected through self-report rather than in-person measurement, so those changes might not be reflected in actual weight loss. However, prior research has consistently reported high correlations between self-reported and objective measures of height and weight (correlation = 0.87–0.98) [58,59,60]. Self-reported weight has been used in a prior large intervention study at a 10-year follow-up [61]. Moreover, the National Health and Nutrition Examination Survey in the U.S. collected self-reported weight data [62]. Nevertheless, future work should collect objectively measured weight data to reduce this potential source of bias. Due to resource constraints, we did not collect quantitative data associated with executive function. However, our qualitative results supported potential intervention-related changes in executive function. For example, participants reported that the intervention helped them stay focused, think outside the box, and view situations from different perspectives. Interpretation of the study outcomes, for example, body weight, dietary intake, and physical activity, warrants caution because of the small sample size in addition to the lack of a control group. We only included women with internet access, which might have created a potential selection bias among participants. Yet, based on a report by the Pew Research Center, 76–85% of low-income individuals own a smart phone [63]. The short intervention with no follow-up limited our ability to assess applications of GoEFT beyond 3 weeks. Even so, the results of our other 20-week intervention applying GoEFT supported that participants enjoyed applying GoEFT [38].

## 5. Conclusions

The GoEFT intervention was acceptable to participants and showed strong preliminary efficacy. Results of the pilot study suggest that GoEFT can be a potentially promising approach to integrate executive function, motivation, and affect to promote weight loss. We have demonstrated the feasibility of applying GoEFT in a real-world setting. Our participants enjoyed the intervention and found it to be helpful beyond weight loss, for example, improving relationships with children. Future large-scale randomized controlled trials are warranted to validate the study findings.

## Figures and Tables

**Table 1:** Examples of semi-structed interview questions.

Think about the second part about choosing a weekly goal from the list we provided (refer to pre-written goal) a. How did you like that? (Probe: give me some examples, be more specific, tell me more, and in what way. Why?). b. How helpful were the goals? (Probe: give me some examples, be more specific, tell me more, and in what way. Why?).
Remember the 5Ws (WHAT, WHY, WHERE, WHEN, WHO) and H (HOW). Tell me three things you liked most about that part of the program (Probe: give me some examples, be more specific, tell me more, and in what way. Why?).
Tell me three things you liked least about the 5Ws and H. (Probe: give me some examples, be more specific, tell me more, and in what way. Why?). a. How can we do better to improve this section?
Let’s talk about the self-care booster (refer to goal progress evaluation) a. How did you like that part? b. What did you like the most about that? c. What else did you like about the “self-care booster?” (Probe: give me some examples, be more specific, tell me more, and in what way. Why?).
How has joining the program affected other areas of your life? (if needed) For example, have you found yourself making more plans in other areas of your life? (Probe: give me some examples, be more specific, tell me more, and in what why. What has been helpful or not helpful and why?)

**Table 2:** Common themes with examples.

Common Themes	Quotes
Helpfulness of Pre-written goals	“Whoever conducted this research study has really put tremendous thought into each goal. It (the pre-written goal) definitely gave me a boost of confidence afterwards… made me like ‘Okay, these are my goals for the week; this is how I’m going to accomplish it”. “I’ve never had to think about my goal and writing… I would just think about in my head…I think it was more useful to see it and writing it because it made it more concrete more real”. “It was a built-in-accountability tool, so that I couldn’t just think about maybe I want to change this, or maybe I want to meal plan or maybe”.
Usefulness of applying 5Ws and HOW to make plans for accomplishing goals	“It (5Ws and How) made me think of an actual plan, not just …like oh I’m going to work out, but instead… okay when, how, who am I going to be with, where, and that was really helpful to visualize how I’m going to do something and then, once I did, that it was easier to actually put into practice”.
Helpfulness of example plans	“I liked that they had very concreate examples of how other women have overcome certain challenges that I’m facing specifically related to like positive self-talk and managing stress”.
Benefits for engaging in goal progress evaluation	“I thought it was good, because it gave me a chance to think about did I actually do the things I wanted to do and the process of how the week went when I was trying to accomplish those goals”.
Increasing motivation to implement plans through GoEFT	“that visualization was really helpful for me” “I like picturing what I wanted and like actually putting it down on paper …this is what I want now, how do I get there, what are the things that I want to do to get there, who like it, it made me think more elaborate about the dream and the things that I want to accomplish in my life” “the most helpful thing …that I’ll definitely carry with me after this (after completing the intervention)”. “I’m picturing the outcomes that I wanted and picturing my family happy and healthy because that’s really the important part”. “mental picture idea that will definitely stick with me and may be useful for a very long time” “I shared the picturing how things go if things went wrong…with my husband and he found it helpful. He was really stressed about something and I like broken down…what the worst things that can happen…what can we do to not make that happen, how we react... that really helped us get through... I found that really helpful”.
Positive intervention impact on children, confidence, and mental health	“It (the intervention) made me think more of me versus always being in mommy mode thinking about us (her and her children). …I can focus more on me and my emotions and how to fix things. I don’t get quite as grumpy and upset with my kids as I did for a while, which is good”. Things are more manageable than I was thinking and that just a little bit of positive self-talk really changes, a whole life” “the positive self talk that’s change, which is tremendously helped my mental tremendously once my mental is good and the rest of the day, seems to go smoothly”. “my response to expectations, not being as hard on myself if I don’t meet goals” “Change the way I think about myself… improved my self-esteem,… I am really happy... I’m also like the steps where it’s like happy healthy family thinking of a happy healthy family and then think about like the pros and cons of. If I continue the cons of if I continued being sad, stress, you know, whatever versus just change it and being happy healthy so that has been really awesome I have also taken that to heart and its really changed my outlook on stressful things because instead of like taking it and then just making it bigger and worse and like drowning I can kind of distance myself and say, yes, it’s stressful but its okay I’m still I’m okay”.

**Table 3:** Results of the pilot intervention (N = 12).

	Mean	SD	95% CI		Cohen’s D	*p* Value
Body Weight (lbs)						
T1	194.1	38.04	169.9	218.3		
T2	191.2	37.70	167.2	215.1		
T2 vs. T1	−2.92	4.23	−5.60	−0.23	−0.69	0.036
BMI (kg/m^2^)						
T1	32.14	5.23	28.81	35.46		
T2	31.58	5.20	28.27	34.89		
T2 vs. T1	−0.56	0.73	−1.02	−0.10	−0.77	0.022
Percentage Energy from Fat						
T1	34.59	4.93	31.46	37.72		
T2	31.17	5.06	27.96	34.39		
T2 vs. T1	−3.42	6.72	−7.69	0.85	−0.51	0.106
Added Sugar (tsp)						
T1	21.07	14.88	11.61	30.53		
T2	12.53	8.32	7.24	17.82		
T2 vs. T1	−8.54	17.88	−19.9	2.82	−0.48	0.126
Fruit/Veg (Cup, Including French Fries)						
T1	4.87	3.42	2.57	7.16		
T2	5.72	3.43	3.41	8.02		
T2 vs. T1	0.85	1.75	−0.32	2.03	0.49	0.138
Fruit/Veg (Cup, Excluding French Fries)						
T1	4.73	3.42	2.44	7.03		
T2	5.55	3.45	3.24	7.87		
T2 vs. T1	0.82	1.82	−0.41	2.04	0.45	0.168
Physical Activity (Weekly MET)						
T1	107.0	249.6	−51.6	265.6		
T2	171.5	390.0	−76.4	419.3		
T2 vs. T1	64.46	504.1	−256	384.7	0.13	0.6664
Perceived Stress						
T1	18.33	6.10	14.46	22.21		
T2	14.67	6.64	10.45	18.88		
T2 vs. T1	−3.67	7.01	−8.12	0.79	−0.52	0.097
Emotion Control (Total Score)						
T1	38.50	8.34	33.20	43.80		
T2	42.58	10.61	35.84	49.33		
T2 vs. T1	4.08	5.74	0.43	7.73	0.71	0.032
Autonomous Motivation: Healthy Eating						
T1	37.58	4.01	35.04	40.13		
T2	40.25	3.22	38.20	42.30		
T2 vs. T1	2.67	3.58	0.39	4.94	0.75	0.025
Autonomous Motivation: Physical Activity						
T1	36.00	5.72	32.37	39.63		
T2	40.50	2.71	38.78	42.22		
T2 vs. T1	4.50	5.79	0.82	8.18	0.78	0.021
Autonomous Motivation: Stress Management						
T1	36.50	4.87	33.41	39.59		
T2	40.00	2.83	38.20	41.80		
T2 vs. T1	3.50	3.97	0.98	6.02	0.88	0.011
Self-Efficacy: Healthy Eating						
T1	19.83	4.90	16.72	22.94		
T2	22.25	4.88	19.15	25.35		
T2 vs. T1	2.42	3.96	−0.10	4.94	0.61	0.058
Self-Efficacy: Physical Activity						
T1	25.33	7.18	20.77	29.89		
T2	29.17	7.41	24.46	33.87		
T2 vs. T1	3.83	8.39	−1.50	9.16	0.46	0.142
General Self-Efficacy						
T1	31.42	5.18	28.13	34.71		
T2	34.83	4.24	32.14	37.53		
T2 vs. T1	3.42	5.88	−0.32	7.16	0.58	0.069

T1 = baseline. T2 = immediately after the 3-week intervention. Cohen’s D = effect size. Shaded areas = the lower score, the better.

## Data Availability

Data are not available for other researchers, because we are actively analyzing data to answer our proposed research questions.
